# Modeling and Validation of Anisotropic Thin-Film Deposition on Cylindrical Substrates for Predictable Resistance Control in MEMS Fabrication

**DOI:** 10.21203/rs.3.rs-8253144/v1

**Published:** 2025-12-09

**Authors:** Aditya Tummala, Francesca Marturano, Giorgio Bonmassar

**Affiliations:** 1 Biomedical Engineering, Paulson School of Engineering and Applied Sciences, Harvard University, Boston, MA 02134, U.S.A.; 2 Department of Radiology, Athinoula A. Martinos Center for Biomedical Imaging, Harvard Medical School/Massachusetts General Hospital, Charlestown, MA 02129, U.S.A.

**Keywords:** Physical Vapor Deposition, Gold Films, Fibers, Wires, Monte Carlo Simulation

## Abstract

Precise control of electrical properties in conductive micro-structures is essential for the performance and reliability of micro-electro-mechanical systems (MEMS). However, the nature of anisotropic physical vapor deposition (PVD), such as electron-beam or thermal evaporation on curved or wire-like substrates, complicates the prediction of thin-film morphology and resulting electrical properties. This study develops and validates a geometrically explicit deposition model describing film growth on cylindrical substrates using a generalized pseudo-Lambertian cosine emission profile. Analytical expressions for local film thickness are derived as functions of deposition time, substrate geometry, and source collimation. Monte Carlo simulations confirm that the model accurately reproduces the deposition profile observed with simulated data $\left(R^{2}=0.99\right)$. A closed-form expression for resistance as a function of deposition parameters was also derived, integrating the Fuchs–Sondheimer and Mayadas–Shatzkes (FS-MS) frameworks to account for thin-film electron scattering and grain-boundary effects. Experimental validation was performed via electron-beam evaporation of gold onto cylindrical glass-core wires, with measured resistances spanning 10Ω to 1kΩ across films **70** to **3000** nm thick. The FS-MS predicted resistances exhibited a Pearson correlation of R=0.983(p<0.001) with empirical measurements, confirming the model’s predictive accuracy. Additionally, this study develops an empirical mathematical model that captures the anisotropic behavior of PVD deposition on cylindrical surfaces. The model enables predictive control of thin-film resistivity in MEMS and bio-MEMS structures and offers a simulation framework that generalizes conventional planar thin-film modeling to complex, three-dimensional microfabrication topographies.

## 1 Introduction

Physical Vapor Deposition (PVD) systems play a crucial role in the fabrication of a wide range of electronic, optical, and micro-electro-mechanical systems (MEMS) applications, as well as in tool manufacturing and decorative coatings. In these systems, materials are vaporized and subsequently condensed onto a substrate, forming thin films with desirable properties such as electrical conductivity, wear resistance, or optical transparency. Depending on the technique, this vaporization may arise from resistive heating of a metal boat in thermal evaporators, a focused beam of excited electrons in electron beam (E-Beam) systems, or ion bombardment within a plasma during sputtering. Among PVD techniques, thermal evaporation and electron-beam deposition offer precise control over anisotropic material fluxes, enabling directional coating on complex geometries. When coupled with photolithography or etching, these methods facilitate the creation of micro- and nano-scale components integral to MEMS architectures, including micro-actuators, sensors, resonators, and biomedical interfaces.

**Fig. 1:**
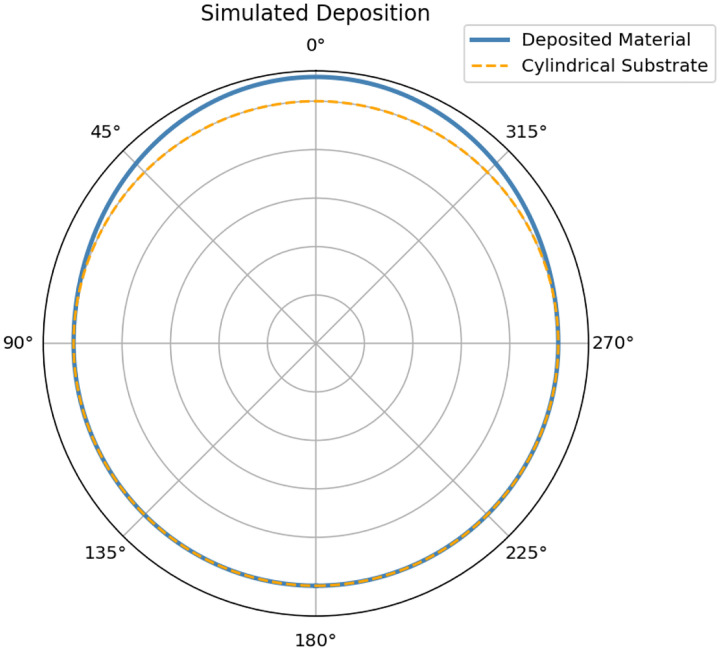
2-D cross section of a simulated deposition on a cylindrical substrate. Emission broadening exponent n=2.

Metallic and composite wires are among the most common substrates used in such applications, functioning as conductive pathways, micro-electrodes, or structural supports. For these components, achieving predictable electrical resistance is critical to ensuring device performance, sensitivity, and signal stability. However, resistance control in thin metallic films remains nontrivial due to the interplay between electron–matter interactions, surface scattering, and morphological variations inherent to the deposition process. In anisotropic PVD systems, deposition occurs primarily along line-of-sight trajectories, leaving shaded or occluded regions undercoated—a significant limitation when working with cylindrical or otherwise non-planar substrates typical in MEMS and bio-MEMS devices (See Figure 1).

Although recent advances in conformal deposition—such as high-pressure PVD (Hass et al. (2004)), hot-wire chemical vapor deposition (Shi (2015); Schropp (2015)), and atomic layer deposition (Yang et al. (2018))—have enhanced coating uniformity, these approaches often necessitate long and costly deposition times, elevated substrate temperatures or employ toxic and expensive precursors, restricting their suitability for bio-compatible MEMS applications. Consequently, despite the broad use of anisotropic PVD in micro-fabrication, existing models rarely capture the true spatial dependence of film growth on curved surfaces or the resulting electrical heterogeneity in deposited traces.

Classical frameworks, such as those proposed by Mayadas and Shatzkes (1970) and Sondheimer (1952), relate film resistivity to micro-structural and scattering parameters; yet, they assume planar geometries and uniform thickness distributions. These simplifications often fail to reproduce empirical resistance measurements in wire-like systems, highlighting the need for a more comprehensive analytical treatment of anisotropic deposition processes.

This study addresses this gap by developing and validating a predictive framework for anisotropic thin-film deposition on cylindrical substrates. The Fuchs–Sondheimer (FS) and Mayadas–Shatzkes (MS) models (Mayadas and Shatzkes (1970); Fuchs (1938); Sondheimer (1952); Jeong et al. (2023)) are integrated with a geometrically resolved deposition model derived from first principles to account for boundary and scattering effects. Finally, this study presents an empirical mathematical model that describes the anisotropic deposition behavior of PVD films on cylindrical surfaces and establishes a foundation for predictive resistance control in MEMS and bio-MEMS fabrication, advancing the predictive design of conducting thin-film architectures. The predicted deposition profile is compared to three-dimensional Monte Carlo simulations, and the analytical resistance estimates are validated with experimental data from electron-beam deposited gold films.

## 2 The Theory

This section establishes a theoretical basis for modeling anisotropic material deposition during E-Beam evaporation onto cylindrical substrates. We first determine the geometric configuration governing the emission and incidence of vaporized particles on a generalizable cylindrical substrate to derive an analytical expression for the local film thickness distribution. This deposition profile is then used to formulate a predictive relationship between the deposition parameters and the resulting electrical resistance of the thin conductive trace.

### 2.1 The Geometry

**Fig. 2:**
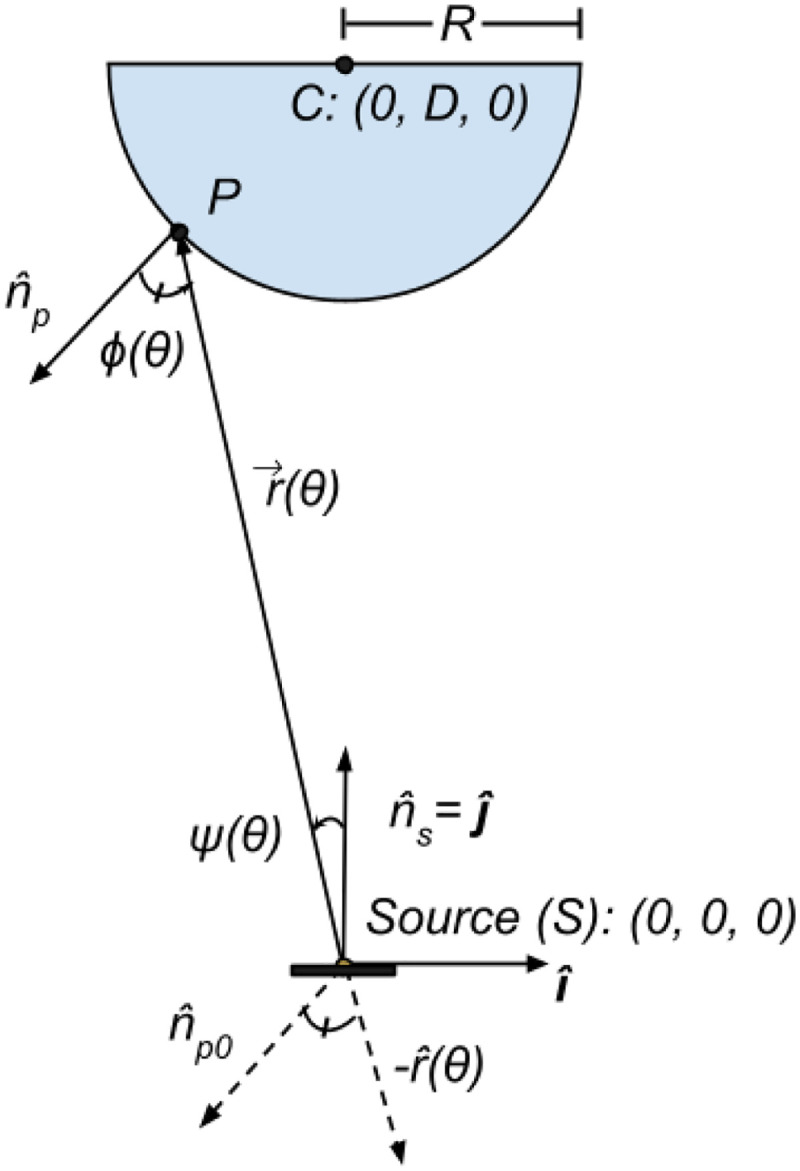
Established geometry for physical vapor deposition processes

The particle emission source is centered on the origin S=(0,0,0). A cylindrical substrate W of radius R about the z-axis is centered at C=(0,D,0), where D is defined as the distance between substrate center C and the emission source S. The cross-section in the x-y plane is shown in Figure 2. The bottom hemisphere of the circular cross–section of the cylinder is defined by

(1) P(θ)=(Rcosθ,Rsinθ+D),θ∈[π,2π].

Since in PVD processes, the sample (substrate) is often positioned above the source material to facilitate uniform coating and to leverage the properties of the vaporized material, deposition only occurs on the bottom hemisphere of W facing the substrate. $\overset{ˆ}{n_{p}}(\theta )$ is the outward unit normal at deposition point P along the surface of the cross section, and $\overset{ˆ}{n_{p0}}(\theta )$ is the same unit normal, but shifted to originate at the emission source, S

(2) $$\overset{ˆ}{n_{p0}}\left(\theta \right)=\overset{ˆ}{i}\;\text{cos}\;\theta +\overset{ˆ}{j}\;\text{sin}\;\theta$$

where $\overset{ˆ}{i}$ and $\overset{ˆ}{j}$ are versors in the x-axis and y-axis directions respectively and $\overset{ˆ}{n_{p}}(\theta )\|\overset{ˆ}{n_{p0}}(\theta )$. The particle emission source occurs at a point at S=(0,0) (a distance D>0 below the center of the substrate cylinder) whereby the outward unit normal to the source is ${\overset{ˆ}{n}}_{s}=\overset{ˆ}{j}$.^1^

A ray of particles from the source S to the substrate at a generalizable point P(θ) can be expressed as

(3) $$\vec{r}\left(\theta \right)=\vec{SP\left(\theta \right)}=\overset{ˆ}{i}R\;\text{cos}\;\theta +\overset{ˆ}{j}(R\;\text{sin}\;\theta +D),\;\theta \in [\pi ,2\pi ]$$

with magnitude:

(4) $$\|\vec{r}(\theta )\|=\sqrt{R^{2}+D^{2}+2RD\;\text{sin}\;\theta }$$

and unit vector:

(5) $$\overset{ˆ}{r}(\theta )=\frac{\vec{r}(\theta )}{\left\|\vec{r}(\theta )\right\|}=\frac{\overset{ˆ}{i}R\;\text{cos}\;\theta +\overset{ˆ}{j}(R\;\text{sin}\;\theta +D)}{\sqrt{R^{2}+D^{2}+2RD\;\text{sin}\;\theta }}$$

Then, the cosine of the emission angle ψ(θ) between $\overset{ˆ}{n_{s}}$ and $\overset{ˆ}{r}(\theta )$ is defined as

(6) $$\text{cos}(\psi (\theta ))=\overset{ˆ}{r}(\theta )\cdot {\overset{ˆ}{n}}_{s}=\frac{\left(R\;\text{cos}\;\theta )(0)+(R\;\text{sin}\;\theta +D)(1\right)}{\left\|\vec{r}\right\|}=\frac{R\;\text{sin}\;\theta +D}{\left\|\vec{r}\right\|}$$

and the incidence angle ϕ(θ) between $\overset{ˆ}{n_{p}}$ and $-\overset{ˆ}{r}(\theta )$

(7) $$\text{cos}(\phi (\theta ))=-\overset{ˆ}{r}(\theta )\cdot {\overset{ˆ}{n}}_{p0}=\frac{\left(-R\;\text{cos}\;\theta )(\text{cos}\;\theta )+(-R\;\text{sin}\;\theta -D)(\text{sin}\;\theta \right)}{\left\|\vec{r}\right\|}=\frac{-R-D\;\text{sin}\;\theta }{\left\|\vec{r}\right\|}$$

**Fig. 3:**
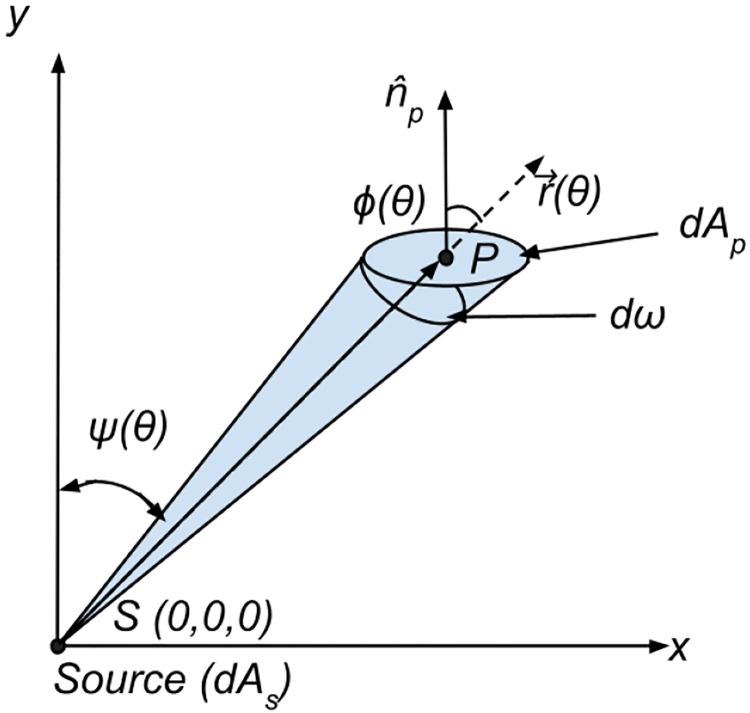
Geometric representation of the differential solid angle dω

To model a differential particle flux onto the differential area of deposition, the property of solid angle must be used, as depicted in Figure 3. Treating the differential deposition area as $dA_{p}$ and the differential source area as $dA_{s}$, we have that the differential solid angle dω[sr], can be described as (Wang et al. (2018))

(8) $$d\omega =\frac{dA_{p}\;\text{cos}(\phi (\theta ))}{\|\vec{r}(\theta ){\|}^{2}}$$

Thus, in the cases when ϕ(θ)=0, (when $\theta =\frac{3\pi }{2}$, See Figure 2), the deposition is maximal when dω reaches its maximum (i.e., when $\phi (\theta )=0,\theta =\frac{3\pi }{2}$).

### 2.2 Deposition Profile

According to this geometric setup, we can describe $d\Phi_{\text{emit}}$ as the differential deposition rate at $dA_{p}$ and ${ℒ}_{s}\left[N_{p}s^{-1}m^{-2}sr^{-1}\right]$ as the particle radiance where $N_{p}$ is defined as the number of particles emitted by the source. Then, as shown in Wang et al. (2018), $d\Phi_{\text{emit}}$ can be expressed as

(9) $$d\Phi_{\text{emit}}\left(\theta \right)={ℒ}_{s}{\;\text{cos}}^{n}(\psi (\theta ))dA_{s}d\omega ,\;\left[\Phi_{\text{emit}}\right]=\left[N_{p}s^{-1}\right]$$

Substituting equation Eq. (8) into Eq. (9), we attain:

(10) $$d\Phi_{\text{emit}}(\theta )={ℒ}_{s}\frac{{\text{cos}}^{n}\left(\psi \left(\theta \right)\right)\;\text{cos}(\phi (\theta ))}{\|\vec{r}(\theta ){\|}^{2}}dA_{s}dA_{p}$$

The local particle deposition density $dJ_{p}(\theta )\left[N_{p}m^{-2}s^{-1}\right]$ is found by dividing Eq. (10) by $dA_{p}$:

(11) $$dJ_{p}(\theta )={ℒ}_{s}\frac{{\text{cos}}^{n}\left(\psi \left(\theta \right)\right)\;\text{cos}(\phi (\theta ))}{\|\vec{r}(\theta ){\|}^{2}}dA_{s}$$

Since the source is simplified as a point emitter, the integration over its area, $A_{s}$, gives the particle deposition density $J_{p}(\theta )$:

(12) $$J_{p}(\theta )=\int_{A_{s}}{ℒ}_{s}\frac{{\text{cos}}^{n}\left(\psi \left(\theta \right)\right)\;\text{cos}(\phi (\theta ))}{\|\vec{r}(\theta ){\|}^{2}}dA_{s}\;=C\frac{{\text{cos}}^{n}\left(\psi \left(\theta \right)\right)\;\text{cos}(\phi (\theta ))}{\|\vec{r}(\theta ){\|}^{2}}$$

where constant $C={ℒ}_{s}A_{s}\left[N_{p}s^{-1}\right]$. The rate of film growth $\dot{f}(t,\theta )\left[ms^{-1}\right]$ is given by multiplying $J_{p}(\theta )$ by the particle volume $V_{p}\left[m^{3}N_{p}^{-1}\right]$. Given $P_{s}(\theta )\in [0,1]$ as the sticking probability, i.e., the probability that an incoming particle adheres to the previously deposited material, and the constant $K=CP_{s}V_{p}\left[m^{3}s^{-1}\right]$ and substituting it into Eq. (12):

(13) $$\dot{f}\left(t^{\prime },\theta \right)=K\frac{{\text{cos}}^{n}\left(\psi \left(\theta \right)\right)\;\text{cos}(\phi (\theta ))}{\|\vec{r}(\theta ){\|}^{2}}$$

Then, f(t,θ)[m] is found by integration,

(14) $$f(t,\theta )=\int_{0}^{t}\dot{f}\left(t^{\prime },\theta \right)dt^{\prime }=Kt\frac{{\text{cos}}^{n}\left(\psi \left(\theta \right)\right)\;\text{cos}(\phi (\theta ))}{\|\vec{r}(\theta ){\|}^{2}}$$

Substituting Eq. (6) and Eq. (7) into Eq. (14),

(15) $$f(t,\theta )=Kt\frac{{\left(\frac{\left(D+R\;\text{sin}\;\theta \right)}{\left\|\vec{r}(\theta )\right\|}\right)}^{n}\left(\frac{\left(-R-D\;\text{sin}\;\theta \right)}{\left\|\vec{r}(\theta )\right\|}\right)}{\|\vec{r}(\theta ){\|}^{2}}\;=-Kt\frac{\left(D+R\;\text{sin}\;\theta {)}^{n}(R+D\;\text{sin}\;\theta \right)}{{\left(R^{2}+D^{2}+2RD\;\text{sin}\;\theta \right)}^{\frac{n+3}{2}}}$$

However, note that ${\text{lim}}_{\theta \to \pi ,2\pi }\;f(t,\theta )<0$. Specifically, this occurs where the term R+Dsin(θ)>0 (i.e. when sin(θ)>-R/D), that is, near the substrate boundaries. Indeed, for θ→π,2π the raw analytic form of the model predicts small negative values near these edges because the cosine projection term allows for slight “overhang” where the surface normal begins to turn away from the source. However, in real physical vapor deposition, these regions simply receive negligible flux Since we assume D≫R, as is with almost all PVD systems, we can simplify the R+Dsin(θ) term to Dsin(θ), which is always negative across θ∈[π,2π]. The deposition at any point P(θ) along the circumference of the cylindrical substrate (fixed R) at time t is therefore given by:

(16) ft,θ=−Kt(D+Rsinθ)nDsinθR2+D2+2RDsinθn+32

### 2.3 Analytical Resistance Model

Eq. (16) provides an expression for evaluating the film thickness f(t,θ) deposited onto a cylindrical substrate surface as a function of time t, deposition geometry (R,D), and the emission broadening factor n. We now extend this model to analytically determine the resulting electrical resistance of the deposited metallic layer, accounting for non-uniform thickness, curvature effects, and thin-film boundary scattering phenomena. To accurately capture electron transport within the thin conductive layer, we employ the Fuchs–Sondheimer (FS) and Mayadas–Shatzkes (MS) frameworks (Fuchs (1938); Jeong et al. (2023)), which correct for grain-boundary and surface-scattering effects that dominate at nanometric scales. During PVD, nanometer-scale film accumulation always occurs at the edges of cylindrical substrates.

At a time t, substrate radius R, and angle θ∈[π,2π], the thickness of deposition is given by Eq. (16). To evaluate the total conductive cross-section formed by the deposited material $A_{cs}(t)\left[m^{2}\right]$, Eq. (16) is integrated over θ from π→2π:

(17) $$A_{cs}\left(t\right)=\int_{\pi }^{2\pi }f\left(t,\theta \right)Rd\theta ,\;\theta \in [\pi ,2\pi ]$$

For typical deposition conditions, the distance from the source to the substrate is significantly larger than the substrate radius (R≪D); thus, $\epsilon =\frac{R}{D}\ll 1$. Substituting Eq. (16) into Eq. (17), setting R=ϵD:

(18) $$A_{cs}(t)=-Kt\int_{\pi }^{2\pi }\frac{D^{n}(1+\epsilon \;\text{sin}\;\theta {)}^{n}(D)(\text{sin}\;\theta )}{{\left(D^{2}\left(\epsilon^{2}+1+2\epsilon \;\text{sin}\;(\theta )\right)\right)}^{\frac{n+3}{2}}}Rd\theta \;=-\frac{KtR}{D^{2}}\int_{\pi }^{2\pi }(\text{sin}\;\theta )(1+\epsilon \;\text{sin}\;\theta {)}^{n}{\left(\epsilon^{2}+1+2\epsilon \;\text{sin}\;\theta \right)}^{-\frac{n+3}{2}}d\theta$$

Expanding to first order in $\epsilon =\frac{R}{D}$, dropping $O\left(\epsilon^{2}\right)$, by the binomial theorem, we have that $(1+x{)}^{a}≃1+ax+O\left(x^{2}\right)+O\left(x^{3}\right)+\ldots$. Applying this to Eq. (18), we expand each term in the integrand:

(19) $$A_{cs}(t)=-\frac{KtR}{D^{2}}\int_{\pi }^{2\pi }(\text{sin}\;\theta )\left(1+n\epsilon \;\text{sin}\;\theta +O\left(\epsilon^{2}\right)\right)\left(-\left(\frac{n+3}{2}\right)(2\epsilon \;\text{sin}\;\theta )+1+O\left(\epsilon^{2}\right)\right)d\theta$$

Substituting $\epsilon =\frac{R}{D}$ in Eq. (19), results in the final expression^2^ for the cross-sectional area of deposition $A_{cs}(t)$:

(20) $$A_{cs}(t)\approx \frac{2KtR}{D^{2}}+\frac{3\pi KtR^{2}}{2D^{3}},\;\left[A_{cs}(t)\right]=\left[m^{2}\right]$$

The first term $\left(\frac{2KtR}{D^{2}}\right)$ is dominant, the second term $\left(\frac{3\pi KtR^{2}}{2D^{3}}\right)$ is a small correction $\left(\propto \frac{R}{D}\right)$, and the rest is negligible (R≪D).

The cross-sectional area of deposition $A_{cs}$, can be used to calculate the predicted resistance of the cylindrical substrate after time t seconds of deposition and a constant volumetric “flow-rate” of material $K\left[m^{3}s^{-1}\right]$. The trace resistance $R_{\text{tot}}[\Omega ]$ is given by:

(21) $$R_{tot}=\frac{\rho_{eff}L}{A}$$

where $R_{\text{tot}}$ is resistance, $\rho_{eff}[\Omega m]$ is the resistivity of deposited material, L[m] is the length of the substrate, and $A\left[m^{2}\right]$ is the cross-sectional area of the substrate.

The resistivity ratio, using the correction for thin metal layers based on the Fuchs-Sondheimer (FS) and Mayadas-Shatzkes (MS) models (Jeong et al. (2023)) to account for boundary and scattering effects in thin films, is given by:

(22) $$\frac{\rho_{0}}{\rho_{\text{eff}}}≃1-\frac{3}{2}\alpha +3\alpha^{2}-3\alpha^{3}\;\text{log}\left(1+\frac{1}{\alpha }\right)-\frac{3(1-P)}{8k}$$

where $\rho_{\text{eff}}[\Omega m]$ depends on the thickness $f(t,\theta ),\rho_{0}[\Omega m]$ is the bulk resistivity of Au, $k=\frac{f(t,\theta )}{l_{0}}\left[mm^{-1}\right]$ (unitless), and P is the fraction of electrons specularly scattered at the external surfaces (Jeong et al. (2023); Mayadas (1968); Tellier and Tosser (1977)). Additionally, $\alpha =\frac{l_{0}r_{gb}}{a_{g}\left(1-r_{gb}\right)}[\Omega m]$ whereby $l_{0}[m]$ is the mean free path of electrons, $r_{gb}[m]$ is the grain boundary reflection constant, and $a_{g}[m]$ is average material crystalline diameter.

Letting $F(\alpha )≔1-\frac{3}{2}\alpha +3\alpha^{2}-3\alpha^{3}\;\text{log}\left(1+\frac{1}{\alpha }\right)$, the FS-MS model becomes:

(23) $$\frac{\rho_{0}}{\rho_{\text{eff}}}≅F(\alpha )-\frac{3(1-P)}{8k}=F(\alpha )-\frac{3(1-P)}{8}\frac{l_{0}}{f(t,\theta )}$$

The longitudinal conductance per unit length G(t) as the parallel sum over the cross-section of the cylindrical substrate^3^ is:

(24) $$G\left(t\right)=\int_{\pi }^{2\pi }\sigma_{eff}\left(f\left(t,\theta \right)\right)f\left(t,\theta \right)Rd\theta ,$$

where the effective conductance $\sigma_{eff}(f(t,\theta ))={\left(\rho_{eff}(f(t,\theta ))\right)}^{-1}$, and thus by Eq. (23),

(25) $$\sigma_{eff}(f(t,\theta ))≃\frac{F(\alpha )}{\rho_{0}}-\frac{3-3P}{8}\frac{l_{0}}{\rho_{0}f(t,\theta )}$$

Substituting Eq. (25) into Eq. (24) to find G(t),

(26) $$G(t)=\frac{F(\alpha )}{\rho_{0}}\int_{\pi }^{2\pi }\underset{A_{cs}}{\underbrace{f(t,\theta )Rd\theta }}-\int_{\pi }^{2\pi }\frac{3-3P}{8}\frac{l_{0}}{\rho_{0}}Rd\theta \;=\frac{1}{\rho_{0}}\left[F(\alpha )A_{cs}(t)-\frac{3-3P}{8}\pi Rl_{0}\right]$$

To account for ambient deposition temperatures $T_{amb}\neq 25^{\circ }C$, in addition to modifications to the temperature-dependent constants listed above, $R_{cyl}$ is adjusted by temperature-dependent constant $T_{adj}≔\left(1+\alpha \left(T_{amb}-25^{\circ }C\right)\right)($Jeong et al. (2023)). Incorporating $T_{adj}$ into Eq. (26) and simplifying, the resistance $R_{cyl}$ on a generalizable cylindrical substrate is

(27) Rcylt=Lcylρ0TadjRFα2KtD21+3πR4D+OR2D2-3-3P8πl0,R≪D

where $R_{cyl}=G(t{)}^{-1},F(\alpha )=1-\frac{3}{2}\left(\frac{l_{0}r_{gb}}{a_{g}\left(1-r_{gb}\right)}\right)+3{\left(\frac{l_{0}r_{gb}}{a_{g}\left(1-r_{gb}\right)}\right)}^{2}-3{\left(\frac{l_{0}r_{gb}}{a_{g}\left(1-r_{gb}\right)}\right)}^{3}\text{log}(1+\left.\frac{a_{g}\left(1-r_{gb}\right)}{l_{0}r_{gb}}\right)$ and $T_{adj}=\left(1+\frac{l_{0}r_{gb}}{a_{g}\left(1-r_{gb}\right)}\left(T_{amb}-25^{\circ }C\right)\right)$.

## 3 Methods

### 3.1 Monte Carlo Simulations

The deposition profile predicted by Eq. (16) was studied using a three-dimensional Monte Carlo simulation developed in Python 3.10 (Google Colab IDE environment) to model particle trajectories originating from a point-approximated evaporation source exhibiting a prototypical pseudo-Lambertian emission profile^4^ $\left({\text{cos}}^{n}(\psi ),n=2\right.,\psi \in \left[-\frac{\pi }{2},\frac{\pi }{2}\right]$). The deposition substrate was represented as a cylindrical wire of 2 cm length $\left(L_{\text{cyl}}\right)$ and 50μm radius (R), with center C positioned coaxially above the source. The source-to-substrate throw distance (D)—a key parameter influencing deposition rate and uniformity—varies widely in literature depending on desired film properties (Yang et al. (2001b); Venkattraman and Alexeenko (2011); Juliannisa et al. (2025)). In this simulation, a throw distance of 5.0 cm was selected, following the optimized configuration reported by Juliannisa et al. (2025), to maximize film uniformity and minimize resistive variability along the cylindrical surface^5^.

A total of 5 billion macro-particles were emitted from the source, of which 1,851,741 were successfully deposited onto the substrate. Each macro-particle corresponded to the deposition of 6.09 × 10^9^ gold atoms, resulting in a maximum film thickness $f_{\text{max}}(t,\theta )\approx 100\;\text{nm}$. This was calculated as an average deposition thickness of the immediate neighboring bins at $P\left(\theta =\frac{3\pi }{2}\right)=\left(R\;\text{cos}\left(\frac{3\pi }{2}\right),R\;\text{sin}\;\left(\frac{3\pi }{2}\right)+D\right)$ at time $t_{\text{max}}$ (region of maximal deposition directly above the source). The simulation took 20 minutes to run (see Supplementary Materials for additional simulation parameters and open-source code). The computed $f_{\text{max}}(t,\theta )$^6^ was determined independently of the simulation bin size, as the raw hit counts were normalized to flux per unit area before thickness conversion. Parameter $Kt\left[m^{3}\right]$ was solved for by setting $f\left(t_{\text{max}},\theta =\frac{3\pi }{2}\right)=100\;\text{nm}$ in Eq. (16).

### 3.2 Empiric Resistance Validation Setup

To validate the analytical model for substrate resistance described by Eq. (27), empirical measurements were conducted on E-Beam evaporated gold films deposited onto cylindrical substrates of various diameters and lengths under controlled vacuum conditions. These experiments aimed to directly compare the predicted resistance values reported by Eq. (27) with resistances measured post-deposition using a two-point ohmmeter and impedance spectroscopy methods.

Gold (Au) was selected for its well-characterized bulk resistivity and widespread use in MEMS manufacturing and circuitry. Cylindrical substrates consisted of glass-core wires (radius 55-300μm, length varying from 1.5 to 43.0 cm), mounted on a substrate holder. The source-to-substrate distance was maintained at D=20.0cm, and deposition was performed under a base pressure between 2 × 10^−6^ and 5.0 × 10^−7^ Torr with ambient deposition temperature $T_{amb}$ of $20^{\circ }C$. Deposition rates, monitored via piezoelectric quartz crystal microbalance, ranged between 1.0 and 3.0 Å/s, with film thicknesses $f_{\text{max}}$ between 70 and 3000 nm (PVD deposition setpoint).

An empirical estimate, referred to as the Empiric Model, was also developed by performing a least-squares regression on the experimental data to determine an empiric scaling constant, 𝒞, that optimally scales the conventional formula for trace resistance Eq. (21),

(28) $$R_{cyl}=𝒞\frac{\rho_{0}L}{A}T_{adj}=𝒞\frac{\rho_{0}L}{TW}T_{adj}$$

where $R_{\text{cyl}}$ is the cylindrical substrate resistance, $\rho_{0}$ is the bulk resistivity of Au, L is the cylindrical substrate length, $T_{adj}$ is the temperature adjustment coefficient (defined above in Eq. (27)), T is the deposition thickness, W is the deposition trace width, and 𝒞 is the empiric constant that optimally scales the conventional trace resistance formula expressed by Eq. (21). Empirically, this represents the “width” of deposition on the wire substrate, as the trace width W is scaled by ${𝒞}^{-1}$. This is done to account for the empiric uncertainty on the trace width of deposition on the substrate, as Eq. (28) assumes uniform deposition thickness T across the entirety of the trace width ${𝒞}^{-1}W$.

Theoretical predictions were computed using the parameters extracted from the deposition geometry (R,D,K,andt) and material constants $\left(\rho_{0},l_{0},a_{g},r_{gb},\text{and}\;P\right)$ taken from literature (see Table 1).

The resistance values measured across n=20 wires were compared to those predicted by the FS-MS adjusted model described by Eq. (27) as well as those predicted by the Empiric Model in Eq. (28). To quantitatively assess the agreement between the analytical FS–MS model predictions, the empirical model predictions, and experimental resistance data, a statistical correlation and least-squares regression analysis were performed with outliers removed.

## 4 Results

### 4.1 Monte Carlo Deposition Profile Validation

Results of the Monte Carlo Simulation are shown in Figure 5. The Monte Carlo simulated deposition thickness profile on a cylindrical substrate was compared to the theoretical deposition profile f(t,θ) predicted by the E-Beam evaporation model Eq. (16). Results of this validation are shown in Figure 6. Final fitting (without Gaussian smoothing) validated the alignment of the theoretical deposition profile to the simulated profile, confirming that the geometric model captures the essential shape and falloff of the measured film thickness around the cylinder. The root mean square error (RMSE) was found to be 3.56418 nm, the mean absolute error (MAE) was 3.22174 nm, and coefficient of determination $\left(R^{2}\right)$ was 0.985204.

### 4.2 Empiric Deposition Profile Validation

Cross-sections of the manufactured cylindrical substrates were prepared using a JEOL Broad Ion Beam Milling Cross Section Polisher and imaged using a Zeiss Gemini 560 Field Emission Scanning Electron Microscope (See Figure 7).

### 4.3 Empiric Resistance Validation

Results of model comparisons to empirically measured resistance values can be found in Table 2, reporting Pearson correlation $(R),R^{2}$, RMSE, and MAE. For the Empiric Model, least squares fitting of Eq. (28) to empiric data yielded ${𝒞}^{-1}W=0.172(2\pi R)$, representing an empirical “deposition trace width” of ≈ 17.2% of the total circumference (2πR) of the cylindrical substrate. Both models indicate strong linear relationships with the measured data, confirming that they accurately capture the overall trend of resistance across a range of varying deposition parameters (See Figure 8).

## 5 Discussion

In this study, we derive, validate, and present both a mathematical model and a simplified empirical model to predict electrical properties of anisotropic conductive traces on cylindrical substrates. The derived geometric deposition profile (Eq. (16)) and the FS-MS electron transport-corrected resistance model (Eq. (27)) jointly explain the behavior of anisotropic PVD on cylindrical substrates, and where measured data deviate from FS-MS and scaled trace model predictions. Monte Carlo simulations were performed to validate that a pseudo-Lambertian plume $\left({\text{cos}}^{n}(\psi )\right)$ coupled to line-of-sight geometry reproduces the measured angular thickness distribution with high fidelity, verifying Eq. (16). A non-uniform thickness profile f(t,θ) was mapped onto a parallel-channel conductance integral and incorporating FS–MS scattering corrections to yield resistance predictions. These model predictions were compared to experimental results across 70–3000 nm films. Further, this experimental data was employed to benchmark a simplified empiric trace approximation model, which captures the $R_{cyl}\propto f_{\text{max}}^{-1}$ relationship with empiric scaling term 𝒞, but misses curvature-induced anisotropy and thin-film scattering, clarifying when rapid estimates suffice and when full FS–MS corrections are necessary. Finally, the emission-shape parameter n and the substrate radius to throw distance ratio R/D were interpreted as practical design knobs for targeting resistance, and outline how additional considerations—angle-dependent sticking probability $P_{s}\in [0,1]$, multilayer stacks, and rotation/planetary fixtures—can serve to narrow the remaining gap between theory and fabrication realities relevant to MEMS and bio-MEMS applications.

### 5.1 Principal Findings

This study presents three key findings with significant implications for MEMS, bio-MEMS, and also non-MEMS electrical component design and fabrication. First, the analytical deposition model derived in Eq. (16) successfully reproduces the spatio-temporal dependence of film thickness on cylindrical substrates under anisotropic PVD. Monte Carlo simulations incorporating 5 × 10^9^ particle trajectories confirmed a close agreement with the predicted deposition distribution $\left(R^{2}=0.985,\text{RMSE}=3.56\;\text{nm}\right)$, demonstrating that the pseudo-Lambertian emission assumption $\left({\text{cos}}^{n}(\psi \right)$, n=2) and geometric formulation capture the essential angular dependence of the deposited flux. Second, by integrating the Fuchs–Sondheimer and Mayadas–Shatzkes (FS–MS) electron-scattering frameworks with this derived geometric model, an electron transport-corrected resistance expression (Eq. (27)) was developed. This formulation quantitatively links deposition parameters of time t, throw distance D, and emission collimation n to effective resistivity and total trace resistance $R_{cyl}$. Comparison with experimentally measured resistances across gold films ranging from 70 to 3000 nm in thickness yielded a strong correlation (Pearson R=0.985,0.983 for the Empiric Model and FS-MS Model, respectively), validating the models’ predictive accuracy for non-planar thin-film systems. Finally, a simplified empirical trace approximation model based on the classic relation $R\propto 1/f_{\text{max}}$ was fitted via least-squares regression to experimental data. While this reduced model captures the general scaling trend, it systematically underestimates the resistance of thinner films due to its neglect of curvature, anisotropic coverage, and surface-scattering effects, resulting in greater error as film thickness f(t,θ) approaches either 0 or ∞.

Together, these results confirm that the combined geometric and FS–MS transport framework provides a physically consistent and experimentally validated model for predicting electrical resistance in anisotropically deposited metallic coatings on cylindrical substrates, offering a foundation for more precise resistance control in MEMS and bio-MEMS fabrication.

### 5.2 Physical Considerations

#### 5.2.1 Anisotropic Plume Parameter n

**Fig. 4:**
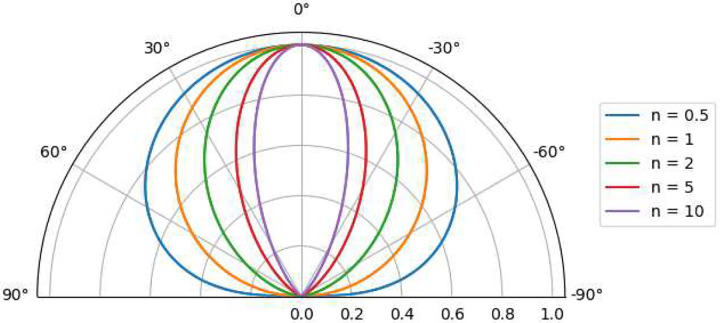
Source emission plume for ${cos}^{n}(\psi )$, modeling various values for n

**Fig. 5:**
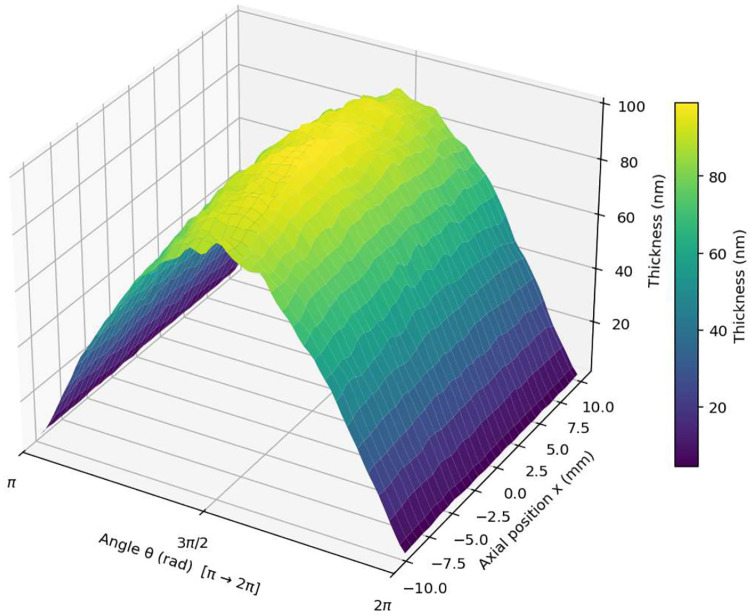
Monte-Carlo Simulation Results

**Fig. 6:**
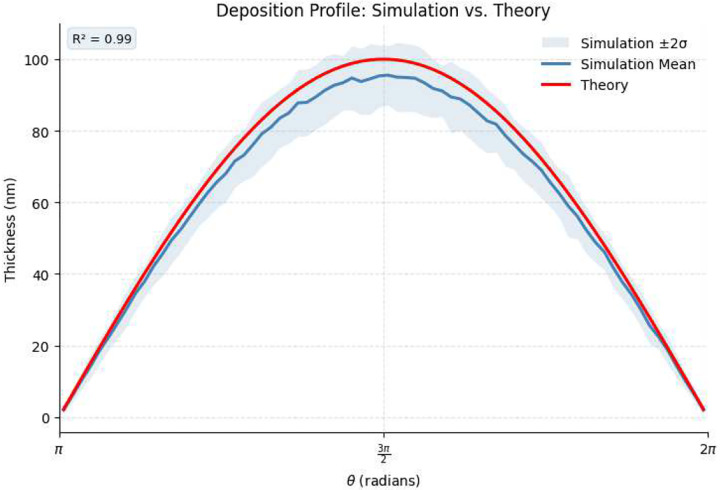
Monte-Carlo simulated data (blue line) vs. theoretical deposition profile (red line). The shaded represents ±2 standard deviations for simulation data.

The emission exponent n in the generalized pseudo-Lambertian term ${\text{cos}}^{n}(\psi )$ governs the angular distribution of vapor flux emitted from the source surface (Yang et al. (2001a); Reinhold et al. (2000)). Physically, n quantifies the collimation or “sharpness” of the emission plume. As portrayed by Figure 4, smaller values (n≈1) correspond to uniform, diffuse emission of particles from the source. In other words, a Lambertian emission profile has equal particle radiance from the source regardless of the viewing angle. Lambertian emission (n=1) is typical of emission from a Knudsen Cell emitter, often used for conformal coating of the interior surface of hollow spherical substrates such as light bulbs and for planetary wafer tooling in vacuum coating equipment (Darling (n.d.)). Pseudo-Lambertian emission (n=2) is typical of thermal evaporation or simpler electron-beam sources, whereas larger values (n>3) describe increasingly forward-directed, collimated plumes that can arise from more directed electron-beam or ion-assisted sources with limited scattering of vaporized particles in the gas phase (Reinhold et al. (2000); Yang et al. (2001a)). In experimental systems, the effective n is influenced by the source-to-substrate throw distance (D), chamber pressure, and the kinetic energy of the evaporated species, all of which alter the degree of angular broadening observed at the substrate (Powell et al. (2000); Iriarte et al. (2011)).

In the derived model for deposition morphology Eq. (16), n directly modulates the falloff of the deposited film thickness f(t,θ) away from the point of normal incidence $\left(\theta =\frac{3\pi }{2}\right)$. Increasing n therefore steepens the angular roll-off and reduces deposition on the lateral and occluded surfaces of the cylinder, effectively narrowing the emission of conductive material onto the substrate. For small substrate lengths, this has little effect on the longitudinal deposition thickness f(t,θ) in the x-direction. However, as the distance from substrate to source becomes smaller or as substrate length becomes longer, n has an increasingly significant effect on overall deposition uniformity and consequent electrical properties.

In practical applications, the effective value of n can be estimated empirically by fitting measured thickness profiles or deposition rates to the analytical form of f(t,θ). For a given material and chamber configuration, n can be calibrated through a simple reference deposition onto a planar witness sample positioned at multiple off-axis angles, as outlined in Fuchs (1938) and Sohn (2019). Once determined, the calibrated n serves as a transferable system parameter for predictive modeling of coating uniformity on curved or wire-like substrates, providing a key input for resistance tuning and process optimization in MEMS fabrication.

#### 5.2.2 FS–MS Corrections

The Fuchs–Sondheimer (FS) and Mayadas–Shatzkes (MS) models are utilized in the derived model for resistance $R_{\text{cyl}}$ (Eq. (27)). Together, these models provide the physical foundation for understanding how thin metallic films deviate from bulk resistivity due to boundary and grain-boundary electron scattering effects (Fuchs (1938); Mayadas and Shatzkes (1970); Jeong et al. (2023)). In bulk conductors, electrons undergo primarily diffusive scattering, and resistivity ρ remains constant with respect to film thickness. However, when the film thickness f(t,θ) approaches or falls below the electron mean free path $l_{0}$, surface and grain-boundary scattering begin to significantly impede carrier motion, causing an increase in effective resistivity $\rho_{\text{eff}}$ relative to the bulk value $\rho_{0}$ (Fuchs (1938); Sondheimer (1952); Mayadas and Shatzkes (1970); Tellier and Tosser (1977)). The FS model accounts for electron reflection at the film surfaces by introducing a specular scattering fraction P, representing the probability that an electron reflects specularly rather than diffusely, while the MS formulation extends this correction to include reflection from grain boundaries parameterized by the reflection coefficient $r_{gb}$ and the mean crystalline grain size $a_{g}$. Both of these effects are incorporated in the FS–MS approximation used in this study (Eq. (23)), yielding a continuous relationship between $\rho_{\text{eff}}$ and f(t,θ).

The influence of FS–MS corrections diminishes as film thickness increases beyond several times $l_{0}$, at which point electron scattering becomes dominated by phonon (representations of quantized lattice vibration) interactions rather than boundary effects (Lacy (2011)). However, in micro-fabrication contexts where thin conductive traces are intentionally deposited at low thicknesses to achieve specific resistances or minimize parasitic coupling, surface and grain-boundary scattering dominate the transport behavior (Islam et al. (2024); Sun et al. (2009)). The strong experimental correlation between measured and FS–MS–predicted resistances (Pearson $R=0.983,R^{2}=0.967$) observed here confirms that these corrections are essential for accurate modeling of non-planar, anisotropic PVD coatings (See Figure 8 and Table 2).

**Fig. 7:**
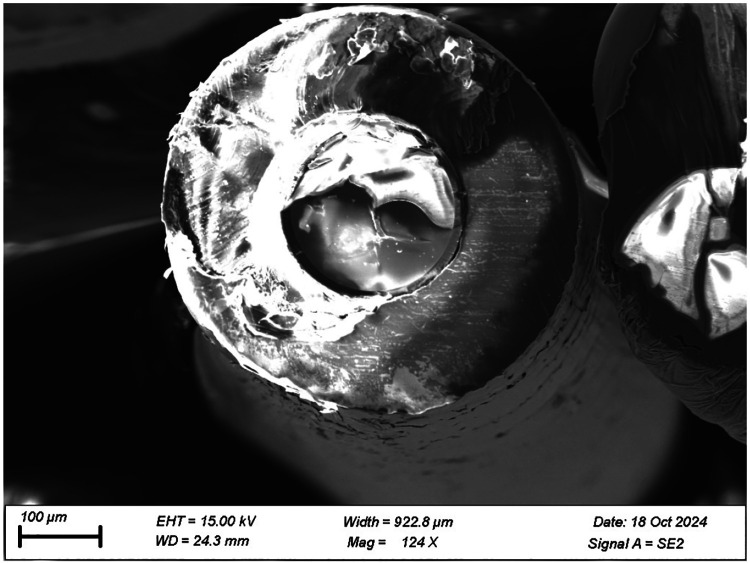
SEM image of substrate cross-section radius 250μm with 1μm Au deposited through electron-beam PVD

Finally, the FS–MS framework also provides an interpretive bridge between morphological observations and electrical performance. SEM imaging (See Figure 7) reveals columnar grain structures and interfacial voids consistent with partial electron reflection $\left(r_{gb}\approx 0.25-0.4\right)$ and low specular scattering (P≈0.1), values aligned with prior measurements of polycrystalline gold films (Zhang et al. (2011); Kästle et al. (2004)). The close quantitative match between these independently derived parameters and the fitted model coefficients underscores the physical robustness of the FS–MS–adjusted resistance formulation, validating its applicability for thin-film systems exhibiting anisotropic deposition, curvature, and micro-structural heterogeneity.

#### 5.2.3 Sources of Discrepancy Between Model and Experiment

While the FS–MS–corrected analytical model (Eq. (27)) reproduces the overall resistance trends with high fidelity (Pearson $R=0.983,R^{2}=0.967$), several systematic deviations between predicted and measured values arise due to additional physical and experimental effects not explicitly included in the present formulation. These discrepancies originate primarily from (1) microstructural evolution during early-stage film growth, (2) mechanical degradation of the deposited layer (i.e., substrate damage and defects), (3) electrical contact variability during measurement, and (4) morphological and geometric effects not captured in the idealized model assumptions.

**Fig. 8:**
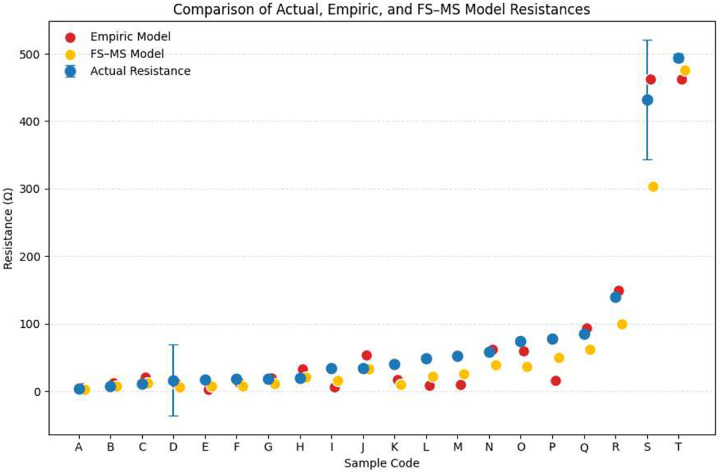
Comparison of measured sample resistance, Empiric Model predictions, and FS-MS Model predictions. 95% CI is plotted for samples with n>1.

**(1) Island formation and percolation thresholds.** In the initial stages of PVD film growth, as described by the Volmer-Weber growth model, gold atoms nucleate into discrete surface islands that coalesce as thickness increases (Volmer and Weber (1926)). Before complete coalescence, these discontinuous films exhibit significantly higher resistivity due to limited percolation pathways and quantum-size effects (Mirigliano et al. (2020); Cho et al. (2025), Morris (2022)). Other models, such as Stranski–Krastanov growth, include the consideration of island formation, while blending theories of conformal deposition and percolation, such as that formalized later on by the Frank-Van der Merwe model of layer-by-layer growth (Stranski and Krastanov (1938); Frank and der Merwe (1949)). The analytical model derived in this work assumes a continuous “layer-by-layer” model of deposition, whereby there exists a conductive layer from the onset of deposition and thus underestimates resistance for films below ~100 nm. This explains the slight upward deviation of experimental resistance from theoretical predictions in the thinnest films (See Figure 8).

**(2) Mechanical cracking and flaking.** SEM imaging (See Figure 7) and post-handling observations revealed local delamination and micro-crack formation along the curved substrate surface. These phenomena arise from tensile stress gradients across the wire substrate and poor adhesion at the gold-glass interface, especially at oblique incidence angles where film density and thickness are reduced, despite a thin 10 nm Ti layer used for adhesion. Such discontinuities interrupt conductive pathways, increasing measured resistance beyond the FS–MS prediction, which assumes a uniform, consistently-adherent film. Similar flaking and cracking-induced resistance jumps have been reported in evaporated thin films of gold and various other common PVD materials on fiber and polymer substrates (Avilés et al. (2009); Mironov et al. (2024); Panjan et al. (2020)).

**(3) Contact and measurement artifacts.** The resistance measurements were performed primarily using a two-point ohmmeter probe, which introduces small but systematic contact resistances at the wire–electrode interfaces. Although minimized by repeated averaging, these effects become proportionally significant in low-resistance samples $\left(R_{\text{cyl}}<10\;\Omega \right)$. Small variations in probe placement, contamination at the gold surface, and probe pressure can further increase apparent resistance, particularly for short-length substrates. Fabrication with robust contacts or Kelvin sensing could mitigate this uncertainty in future work.

**(4) Geometric simplifications and unmodeled effects.** As discussed in Section 2, the theoretical model treats the evaporation source as a perfect point emitter and neglects secondary scattering in the vapor phase, assuming entirely free molecular flow. In practice, however, non-uniform angular flux and evaporation crucible or boat source area produce subtle shifts in the peak deposition angle and broaden the film distribution compared to the idealized ${\text{cos}}^{n}(\psi )$ profile. Additionally, substrate curvature, rotation, micro/nano defects, and surface roughness can locally modify the incidence angle ϕ(θ) and sticking probability $P_{s}$, introducing small spatial variations in film thickness f(t,θ) not captured analytically. These combined geometric effects yield slight asymmetries in the measured film thickness around the cylindrical substrate and contribute to residual variance in the experimental data (Yang et al. (2001b); Venkattraman and Alexeenko (2011); Juliannisa et al. (2025)).

Collectively, these effects highlight the major considerations in assuming perfect continuity, adhesion, and isotropic electron transport in thin metallic coatings. Nonetheless, the residual error between the model and experiment remains well within experimental uncertainty, confirming that the dominant behavior of anisotropic PVD on cylindrical substrates is accurately described by the combined geometric and FS–MS theoretical framework.

### 5.3 Experimental Constraints and Approach

While the theoretical expressions of Eq. (16) and Eq. (27) assume idealized boundary conditions, their application to real-world PVD systems necessitates controlled simplifications to achieve both computational tractability and experimental reproducibility. The following subsections outline the key methodological considerations, parameter sensitivities, and experimental constraints that shaped the design, validation, and interpretation of results.

#### 5.3.1 Monte Carlo Methodological Remarks

The Monte Carlo simulation framework developed for this study was designed to isolate purely geometric and angular effects in anisotropic deposition, independent of material-specific phenomena such as re-emission, surface diffusion, or temperature and incident angle-dependent sticking probability. Each emitted macro-particle was assigned an initial direction drawn from the normalized pseudo-Lambertian distribution ${\text{cos}}^{n}(\psi )$, with n=2. This configuration reflects the typical emission profile of E-Beam evaporation sources operating under high vacuum (< 10^−6^ Torr), where we assume free molecular flow and gas-phase scattering is negligible (Yang et al. (2001b); Venkattraman and Alexeenko (2011)).

A total of 5 × 10^9^ particle trajectories were simulated to ensure a statistical convergence of the deposited flux profile on the cylindrical substrate. Rather than interpreting raw hit counts in each surface bin directly as thickness, the tally in each bin was converted to a flux density by normalizing each bin with respect to both the local deposition area and the corresponding solid angle subtended at the source. In other words, each bin recorded particles per unit area per unit solid angle, so that changes in the number or size of bins do not alter the recovered angular thickness profile. This area and solid-angle normalization prevent spatial discretization artifacts that would otherwise arise from uneven bin sizes or coarse angular sampling, ensuring that the simulated thickness distribution remains invariant under mesh refinement. These bin-independent flux estimates are essential for making meaningful, bin resolution-agnostic comparisons between the Monte Carlo results and the continuous analytic solution for deposition thickness f(t,θ).

The chosen throw distance of D=5.0cm and cylinder radius of R=50μm correspond to an optimal practical geometry used in small-scale MEMS tooling and fiber metallization systems (Juliannisa et al. (2025); Yang et al. (2001b); Venkattraman and Alexeenko (2011)). This radius-to-distance ratio (R/D=0.001) ensures that line-of-sight deposition dominates, consistent with the small-angle approximation used in deriving Eq. (16). The Monte Carlo model thus serves primarily to validate the angular dependence and geometric fidelity of the analytical framework rather than to replicate detailed atomic transport or post-deposition grain percolation. Future refinements may incorporate rotational motion, substrate heating, or diffusive re-emission effects to further approximate the physical PVD environment.

#### 5.3.2 Experimental Constraints and Imaging Challenges

Empirical validation of thin-film thickness and morphology on cylindrical substrates presents several technical challenges that inherently limit the precision of comparison between model and experiment beyond simple resistance comparison. Unlike planar substrates, wires or fiber-like samples are difficult to cross-section cleanly without introducing mechanical deformation or fracture. Indeed, even minor cutting artifacts can distort the apparent thickness or cause delamination at the metal–substrate interface. To minimize these effects, cross-sections were prepared using broad ion-beam (BIB) milling and imaged via field-emission scanning electron microscopy (FESEM), providing near-vertical cuts (See Figure 7). Nevertheless, curvature-induced charging and sample tilt occasionally limited image clarity in the peripheral regions, particularly at large θ. Additionally, the ratio of deposition thickness to substrate radius $f_{\text{max}}/R$ is very small. Both achieving large thicknesses and using extremely fine substrates (R<10μm) are resource-intensive, time-intensive, and overall highly impractical, especially with gold films. Therefore, visual measurements of the cross-section to characterize the deposition profile tend to be extremely inconsistent and non-viable.

Despite these challenges, however, the combination of analytical modeling, high-fidelity simulation, electrical measurements, and careful imaging provided sufficient quantitative agreement to validate the theoretical framework. The results demonstrate that even under non-ideal experimental conditions, the anisotropic deposition behavior and its impact on electrical performance can be accurately captured through geometric modeling, properly calibrated FS–MS corrections, and controlled measurement methodology.

### 5.4 Applications

Beyond validating the physical accuracy of the analytical and FS–MS–corrected model frameworks, the results of this study have direct implications for the design and optimization of conductive thin-film structures in microscale and biomedical device fabrication (MEMS and bio-MEMS), as well as other micro-electronic use cases. The developed models establish a set of scalable, analytical tools that connect geometric deposition parameters to measurable electrical outcomes, thereby enabling predictive control of resistance and film morphology across diverse PVD configurations.

#### 5.4.1 Model Applicability

The proposed model is broadly applicable to anisotropic PVD environments in which film growth is dominated by line-of-sight vapor flux and negligible scattering in the vapor phase (assumption of free molecular flow). This includes PVD processes like electron-beam evaporation, thermal evaporation, and certain ion-beam-assisted deposition (IBAD) systems under high vacuum (< 10^−6^ Torr) (Mattox (2010); Panjan et al. (2020)). The geometric foundation expressed in Eq. (16) remains valid as long as the substrate curvature satisfies R/D≪1, ensuring that occlusion and self-shadowing effects can be modeled through the ${\text{cos}}^{n}(\psi )$ and cos(ϕ) angular factors without resorting to full ray-tracing or molecular dynamics simulation. For most MEMS-scale or fiber-based devices, where substrate diameters range from tens to hundreds of micrometers, this condition is easily met for typical PVD throw distances of 5–30 cm. However, for use-cases in which R/D is not small, Eq. (27) must be modified to account for error factors beyond $O\left(\epsilon^{2}\right)$, where we recall ϵ=R/D. See Supplementary Materials for more details.

Because the model incorporates surface and grain-boundary scattering through the FS–MS framework, it extends naturally to other metals and alloys used in microelectronic applications. By substituting appropriate material constants—bulk resistivity $\rho_{0}$, mean free path $l_{0}$, grain size $a_{g}$, and reflection coefficients $r_{gb}$ and P—the model can predict electrical performance for Ag, Cu, Al, Ti, Pt, and Ni films. For example, copper and silver, which exhibit higher specular scattering fractions (P≈0.3-0.4) and longer mean free paths, are expected to show a weaker thickness dependence of resistivity than gold, while refractory metals such as Ti or Pt may exhibit stronger deviations due to increased grain-boundary scattering and smaller $a_{g}$ (Jeong et al. (2023); Zhang et al. (2011), Glushko and Dehm (2019)). These variations can be quantified directly through the substitution of material parameters in Eq. (27), making the framework compositionally generalizable.

Specifically, although this study focuses on metallic thin films, specifically thin gold films, the geometric deposition formalism and analytical model for $R_{\text{cyl}}$ remain valid for dielectric, semiconducting, and even non-conductive coatings such as ceramic deposited under anisotropic PVD conditions (Tyunkov et al. (2022); Oks et al. (2017)). For dielectric materials such as SiO_2_, TiO_2_, or Al_2_O_3_, the same angular flux distribution governs local film thickness, though the electrical behavior shifts from resistive to capacitive or insulating. In such cases, Eq. (16) still provides the spatial dependence of film thickness necessary for modeling dielectric breakdown strength, leakage pathways, or optical interference effects in cylindrical geometries (Kameshkov and Gerasimov (2024)). Similarly, when considering layered or composite metallizations such as Ti/Au or Cr/Au stacks, inter-layer diffusion and adhesion layers modify effective scattering parameters, introducing additional interfaces that can be modeled through series or parallel resistance combinations (Todeschini et al. (2017)). Thus, the model provides a versatile foundation for expanding beyond pure-metal systems to composite and functional thin films.

#### 5.4.2 Implications for MEMS and Bio-MEMS

The predictive relationship between deposition geometry, film thickness, and electrical resistance established in this work holds significant implications for the design of MEMS and bio-MEMS devices that rely on precise conductive pathways. For micro-fabricated sensors, resonators, and micro-actuators, resistance uniformity directly influences signal stability, thermal dissipation, and response time (Jeong et al. (2023); Cheng et al. (2025)). The derived analytical form of $R_{\text{cyl}}(t)$ enables engineers to pre-compute resistance values for given deposition parameters, minimizing the need for iterative fabrication trials.

In bio-MEMS and neural interface applications, the use of cylindrical or fiber-based geometries is quite common. For example, these substrates are used in fabricating micro-electrode arrays, catheter-embedded sensors, and neural stimulation leads (Jeong et al. (2023); Zaidi et al. (2025)). For such systems, the ability to predict resistive behavior as a function of deposition time and geometry allows for finer control over signal impedance, heating, conductance, and biocompatibility. Because the model is purely geometric in its core form, it can be adapted to biocompatible metals such as platinum, iridium, or titanium nitride (TiN) simply by substituting material constants, facilitating safer and more predictable device performance in vivo.

Furthermore, the anisotropic nature of PVD described by this framework can be leveraged intentionally for functional design. By tuning the orientation of the substrate, selective metallization can be achieved along exposed cylindrical regions while shadowed areas remain uncoated. This can be taken further, where differing layer ratios with multi-material stack-based deposition can result in differing, predictable, and tunable electrical properties throughout the wire or fiber substrate. In sum, the presented model serves not only as an interpretive tool for existing deposition processes but also as a predictive design framework for next-generation MEMS and bio-MEMS architectures requiring spatially controlled conductive coatings.

### 5.5 Limitations and Future Study

While the presented framework successfully integrates geometric, transport, and empirical modeling to predict resistive behavior in anisotropically deposited thin films, several simplifying assumptions constrain its quantitative generality. These limitations, in addition to those discussed in Section 5, primarily arise from the idealizations necessary for analytic tractability and the practical constraints of experimental validation.

**(1) Point-source and Vacuum Idealization:** The deposition model assumes a point-like emission source and negligible gas-phase scattering (free molecular flow assumptions), implying that all particle trajectories follow direct line-of-sight paths. In practice, however, as discussed in section 5.3, thermal and electron-beam evaporators exhibit finite source areas and partially diffusive scattering, especially under weaker vacuum environments (> 10^−5^ Torr). This can broaden the angular flux distribution and slightly shift the deposition maximum, leading to underestimation of coating uniformity for short throw distances (D<5cm) or larger substrates (R/D>0.01) (Panjan et al. (2020); Juliannisa et al. (2025)). Future refinements may incorporate extended-source geometry and partial-pressure corrections to more accurately model weak or intermediate-vacuum regimes without a free molecular flow assumption.

**(2) Constant Emission and Material Parameters:** The derived model treats the emission exponent n, sticking probability $P_{s}$, and deposition rate constant K as temporally invariant. However, as the source temperature, chamber pressure, and deposition rate fluctuate during deposition, these parameters can vary dynamically, altering film growth rate, granular geometry, film percolation, and angular uniformity (Mattox (2010); Iriarte et al. (2011)). Similarly, the FS–MS parameters $\left(P,r_{gb},a_{g}\right.,\text{and}\;l_{0})$ are considered uniform and constant across the film thickness and surface. In reality, grain size and specularity evolve during film coalescence, particularly below 200 nm, introducing local heterogeneity not captured by the static model (Zhang et al. (2011); Glushko and Dehm (2019)).

**(3) Omission of Thermal, Stress, and Diffusion Effects:** The model developed in this study neglects the influence of non-ambient substrate temperature, interfacial stress, and atomic diffusion on film microstructure. Indeed, these processes affect adhesion, grain growth kinetics, and mechanical stability, which in turn affect electron scattering behavior, leading to deviation of measured resistance from model predictions. Gold-on-glass systems, for instance, exhibit temperature-dependent stress relaxation that can induce cracking or delamination at low thicknesses (Hutchinson (1996); Leib and Thompson (2010)). Including coupled thermo-mechanical modeling or stress-dependent scattering terms could improve the model’s predictive fidelity for multi-material, multi-layer, or high-temperature depositions.

**(4) Limited Angular Resolution and Measurement Uncertainty.** Empirical characterization of f(t,θ) and $R_{cyl}$ is constrained by the precision of cross-sectional imaging and two-point resistance measurements. The curved geometry of wire substrates introduces systematic uncertainties in angular mapping and local thickness determination, especially near the occluded regions (θ≈π,2π). Measurement artifacts, contact resistance, and film cracking may introduce small but persistent biases that limit direct one-to-one correspondence between simulation and experiment. Incorporating four-point probe testing, in-situ monitoring, or automated angular profilometry could reduce these uncertainties in future work.

**(5) Lack of Dynamic Growth and Rotation Modeling.** The current model framework assumes static deposition without substrate motion. In practical PVD systems, substrate rotation and planetary tooling are frequently employed to improve uniformity, including the wire substrates tested in this study. These dynamic processes effectively average the angular dependence of f(t,θ) and longitudinal deposition dropoff, complicating closed-form solutions for analytical models. Extending the model to rotational or time-dependent boundary conditions would allow direct prediction of uniformity and resistance under standard industrial configurations (Reinhold et al. (2000); Jeong et al. (2023); Zaidi et al. (2025)).

Despite these limitations, the close agreement between theoretical and empirical results across three orders of magnitude in resistance demonstrates that the dominant physical processes, i.e., the anisotropic flux geometry and boundary-limited electron scattering, are accurately captured. The framework thus serves as a reliable, first-principles baseline that can be progressively refined to incorporate dynamic, multi-physical, or stochastic phenomena as required for future MEMS and bio-MEMS design.

## 6 Conclusions

This study developed and experimentally validated an analytical framework for predicting thin-film deposition morphology and electrical resistance in anisotropic physical vapor deposition (PVD) on cylindrical substrates. By coupling a derived first-principles geometric deposition model with Fuchs–Sondheimer and Mayadas–Shatzkes electron-scattering corrections, a closed-form resistance expression was derived that accurately reproduced both Monte Carlo simulations and measured resistances across 70–3000 nm gold films. The model captures how geometric parameters—throw distance (D), curvature (R), and emission collimation (n)—govern spatial variations in film thickness and conductivity, while deviations between prediction and experiment arise primarily from micro-structural and mechanical effects inherent to thin-film growth. This framework generalizes to various materials, multilayer electrical structures, and deposition modalities, providing a practical foundation for predictive control of electrical properties with cylindrical substrates. This work offers a predictive design tool for ensuring consistency and efficacy throughout design, iteration, and fabrication of MEMS and bio-MEMS devices.

## Supplementary Material

Supplementary Files

This is a list of supplementary files associated with this preprint. Click to download.

- SupplementaryMaterials.tex
- DMESupplementaryMaterials.pdf

Supplementary materials are available online, and the code developed for this study is available on GitHub.

## Figures and Tables

**Table 1:** Established Constants for Au

Parameter	Variable	Established Value
Bulk Resistivity^1^	$\rho_{0}$	$2.44\times 10^{-8}[\Omega m]$
Temperature Coefficient of Resistance^1^	TCR	$3.4\times 10^{-3}\left[{{}^{\circ}C}^{-1}\right]$
Electron Mean Free Path^2^	$l_{0}$	$3.0\times 10^{-8}[m]$
Grain Boundary Reflection Constant^3^	$r_{gb}$	0.25 – 0.4
Material Crystalline Size^3^	$a_{g}$	$4.0\times 10^{-8}-3.2\times 10^{-7}[m]$
Specular Scattering Fraction^4^	P	0.1

Note: At 25 °*C*. Values vary for each deposition system and process—the above values are established as generalized values for these constants and may not be accurate for every PVD system.

1 Lloyd (1998)

2 Karna et al. (2023)

3 Zhang et al. (2011); Glushko and Dehm (2019)

4 Kästle et al. (2004)

**Table 2:** Predictive performance of two models against measured resistance (n=20). Values reported: Pearson correlation (R), coefficient of determination $\left(R^{2}\right)$, root mean square error (RMSE), and mean absolute error (MAE).

Model	Pearson R	Pearson p	$R^{2}$	RMSE	MAE
Empiric Model	0.985	< 0.001	0.971	22.38	18.92
FS-MS Model	0.983	< 0.001	0.967	23.74	15.62

## Footnotes

1 Note: This geometric setup is defined so that film growth occurs in the negative $\overset{ˆ}{ȷ}$ direction, analogously to experimental PVD systems.

2 See Supplementary Materials for further details on derivation.

3 See Supplementary Materials for more details.

4 Figure 4 shows the effect of the emission broadening exponent n discussed here.

5 Though a throw distance of 5 cm was selected based on insights gained from Juliannisa et al. (2025), as long as R≪D (i.e. R/D≪1), differences in the values for D makes minimal impact on the final deposition profile

6 $f_{\text{max}}(t,\theta )$ is the deposition setpoint for PVD systems, as measured by a piezoelectric quartz crystal microbalance.
